# Improved Analytical Approach for Determination of Tropane Alkaloids in Leafy Vegetables Based on µ-QuEChERS Combined with HPLC-MS/MS

**DOI:** 10.3390/toxins14100650

**Published:** 2022-09-20

**Authors:** Lorena González-Gómez, Sonia Morante-Zarcero, Jorge A. M. Pereira, José S. Câmara, Isabel Sierra

**Affiliations:** 1 ESCET—Escuela Superior de Ciencias Experimentales y Tecnología, Departamento de Tecnología Química y Ambiental, Universidad Rey Juan Carlos, C/Tulipán s/n, 28933 Móstoles, Madrid, Spain; 2 CQM—Centro de Química da Madeira, Universidade da Madeira, Campus da Penteada, 9020-105 Funchal, Portugal; 3 Departamento de Química, Faculdade de Ciências Exatas e da Engenharia, Universidade da Madeira, Campus Universitário da Penteada, 9020-105 Funchal, Portugal

**Keywords:** atropine, scopolamine, tropane alkaloids, µ-QuEChERS, HPLC-MS/MS, leafy vegetables

## Abstract

This work presents an optimized methodology based on the miniaturization of the original QuEChERS (μ-QuEChERS) followed by liquid chromatography coupled to mass spectrometry (HPLC-MS/MS) for the determination of tropane alkaloids (TAs), atropine, and scopolamine in leafy vegetable samples. The analytical methodology was successfully validated, demonstrating quantitation limits (MQL) ≤ 2.3 ng/g, good accuracy, and precision, with recoveries between 90–100% and RSD ≤ 13% for both analytes. The method was applied to the analysis of TA-producing plants (*Brugmansia versicolor*, *Solandra maxima,* and *Convolvulus arvensis*). High concentrations of scopolamine were found in flowers (1771 mg/kg) and leaves (297 mg/kg) of *B. versicolor*. The highest concentration of atropine was found in flowers of *S. maxima* (10.4 mg/kg). Commercial mixed leafy vegetables contaminated with *B. versicolor* and *S. maxima* were analysed to verify the efficacy of the method, showing recoveries between 82 and 110% for both analytes. Finally, the method was applied to the analysis of eighteen samples of leafy vegetables, finding atropine in three samples of mixed leafy vegetables, with concentrations of 2.7, 3.2, and 3.4 ng/g, and in nine samples with concentrations ≤MQL. In turn, scopolamine was only found in a sample of chopped Swiss chard with a concentration ≤MQL.

## 1. Introduction

Currently, the control of natural toxins in food constitutes a great concern for consumers, food-sector institutions, and governmental entities responsible for the supervision of food quality and safety. Alkaloids—specifically, tropane alkaloids (TAs)—are a family of natural toxins that are being extensively studied due to the risks associated with the consumption of food contaminated with these substances. This family, made up of more than 200 secondary metabolites, appears in numerous families of plants that grow among crops, contaminating them [1]. The Solanaceae family is one of the families with the largest number of TA-producing species, the most relevant being *Atropa belladonna*, *Datura stramonium*, and *Brugmansia*. Even so, TAs also appear in other families such as Brassicaceae, Erythroxylaceae, or Convolvulaceae [2], which have been less studied. For example, the *Convolvulus* species (bindweed) are a problem in European fields, and the European Food Safety Authority (EFSA) recommends studying these species [3]. In 2021, a new regulation of the European Union (EU) 2021/1408 [4] emerged to control two of the most outstanding TAs, atropine and scopolamine. According to this document, different foods must be controlled, such as the ones based on processed and unprocessed cereals, herbal infusions, or cereal-based baby foods. Until now, there have not been many studies focusing on other types of food, such as leafy vegetables and other plant-based foods. However, recently an alert on the RASFF portal [5] in deep-frozen spinach puree showed contamination by atropine and scopolamine in concentrations greater than 1000 ng/g. Also, Castilla-Fernández et al. [6] indicate that the contamination of spinach products by TAs can be due to the similarity with *Datura innoxia* leaves, and concentrations between 0.02–8.19 ng/g were found in the analysed samples. On the other hand, Mulder et al. [3] studied TAs in cereal-based, ready-to-eat meals for children containing vegetables, mixed vegetable stir-fry, and mixed vegetable products such as bell pepper, potato, courgette, onion, cauliflower, cabbage, peas, green beans, broccoli, and carrot. In these foods, higher concentrations of TAs other than atropine and scopolamine were found. Considering the mentioned works, more data are necessary about the presence of TAs in vegetables.

For the analysis of TAs, the sample preparation step plays a very important role. The foods where these types of toxins appear are very complex matrices that can make analysis difficult and prone to some interferences. In addition, the EU recommendations suggest that the methods should achieve low limits of quantification of up to 5 ng/g in the case of agricultural products, ingredients, food supplements, and infusions [7]. Therefore, the sample treatment must be effective to eliminate interferences and matrix effects, which can make reaching these limits a challenging task. Solid-phase extraction and the QuEChERS (quick, easy, cheap, effective, rugged and safe) procedure are the most widely used techniques for the extraction and purification of TAs [2]. However, these techniques use a large volume of organic solvents and generate a high number of residues, which may cause some environmental concerns. For this reason, the field of analytical chemistry is currently tending toward miniaturized methodologies that enable the objectives of green analytical chemistry (GAC) [8,9]. Currently, there are not many works using microextractive techniques in the determination of TAs in foods. To the best of our knowledge, such an approach was only very recently reported, involving a variant of micro-solid-phase extraction (µ-SPE), named µSPEed, for the extraction of atropine and scopolamine in herbal infusions and teas [10]. Similar works can be found in the literature but involve drug or biological samples. For example, µ-SPE has been applied to determine cocaine and cocaethylene in plasma [11], solid-phase microextraction (SPME) for the analysis of drugs, including atropine [12], and the single-droplet, liquid-phase microextraction (SDLME) technique to concentrate scopolamine from hair samples [13]. In this sense, the application of miniaturized techniques in the analysis of TAs in foods is an unexplored field. The original QuEChERS procedure [14] is fast, cheap, and effective and has been applied to extract and purify TAs from food samples such as honey [15,16], animal products [17], cereals [18,19], spinach-based products [6], or teas [20]. However, until now, miniaturization of QuEChERS (µ-QuEChERS) for TAs determination has not been reported. Therefore, the objective of this work was to optimize and validate a more sustainable methodology based on µ-QuEChERS extraction combined with liquid chromatography coupled to mass spectrometry (HPLC-MS/MS) for the determination of atropine and scopolamine in commercial leafy vegetables. In addition, the methodology developed was applied to determine the content of atropine and scopolamine in TA-producing plants as well as in a salad sample intentionally contaminated with *Brugmansia versicolor* and *Solandra maxima*. As far we may know, it is the first time that a miniaturized methodology based on the QuEChERS procedure is applied to the determination of TAs in leafy vegetable samples.

## 2. Results and Discussion

### 2.1. Optimization of the μ-QuEChERS

The current trend in sustainable and environmentally friendly methodologies led us to consider the optimization of the original QuEChERS protocol proposed by Anastassiades et al. [14]. Accordingly, the amounts of salts and solvents proposed in the original protocol [14] were reduced 10 times and the proportions of citrate buffer were adjusted from the work of Izcara et al. [21]. To reduce the amount of sample, the recommendations of the QuEChERS CVUA Stuttgart guide were followed [22]. First, a mixed salad sample with different types of leafy vegetables was chosen for the optimization process (Mix-1). These samples have a moisture content greater than 80%, and so, according to the guide [22], 10 g of the sample should be weighed. Therefore, to achieve miniaturization, the sample amount was reduced 10 times and only 1 g of the fresh and crushed sample was weighed in a falcon tube. On the other hand, for comparative purposes and to obtain homogeneous samples, the Mix-1 sample was freeze-dried. Lyophilization allowed proper sample storage, avoiding their degradation. In addition, by removing the water, a more homogeneous and representative sample can be achieved, and problems in the extraction of TAs caused by the different water content of the leafy vegetable samples are avoided. The water lost after lyophilization was around 90%, and for this reason, only 0.1 g of lyophilized sample was used for the application of the μ-QuEChERS protocol. Subsequently, the μ-QuEChERS protocol was applied to the fresh (1 g) and the lyophilized (0.1 g) samples, being the lyophilized samples previously hydrated with 0.9 mL, 0.75 mL, and 0.5 mL of water to normalize the extraction conditions. To calculate recoveries, two samples were spiked at 5 ng/g at the beginning of the protocol and one sample at the end. Recoveries of 73 ± 3% for atropine and 77 ± 9% for scopolamine were obtained in the fresh sample (Table 1). For the lyophilized samples, large differences were observed between hydrating the sample with 0.9 mL, 0.75 mL, and 0.5 mL. As the mixed leafy vegetable samples contain different types of lettuce and other leafy vegetables, the water content can vary, and for this reason, different amounts of water were tested to hydrate the sample before the μ-QuEChERS protocol. Lower recoveries were shown for 0.9 mL: <70% in both analytes (Table 1). The reason for these lower recoveries may be due to the fact that the content of water (0.9 mL) and ACN (1 mL) is very similar. This can cause the dissolution of the TAs in the aqueous phase, since they are highly soluble compounds in water and polar solvents [2]. By reducing the water content in the hydration to 0.75 and 0.5 mL, better recoveries, close to 100%, were achieved (Table 1). Overall, 0.5 mL of water was selected since lower variations between assays were observed by comparison with 0.75 mL.

To verify if good recoveries could be obtained at higher TA concentrations using 0.5 mL of water, a spiking test with 100 ng/g was carried out, and recoveries percentages of 84 ± 1% for atropine and 105 ± 13% for scopolamine were obtained (Table 1).

The injection solvent for the HPLC-MS/MS analysis was also optimized. For this, the previously optimized conditions, 0.1 g of lyophilized sample and 0.5 mL of water for sample hydration, were considered. The protocol was carried out on two samples spiked at the beginning of the process and one at the end, with 5 ng/g. The solvents considered to redissolve the sample were methanol (MeOH), water, MeOH-water (50:50, *v*/*v*), ACN, ACN-water (50:50, *v*/*v*), water (0.1% formic acid, FA), and ACN-water (both with 0.1% FA, 10:90 *v*/*v*). Figures S1 and S2 show the chromatograms of the mentioned tests. Samples redissolved in MeOH/water (50:50, *v*/*v*), and ACN/water (50:50, *v*/*v*) show larger peaks and better limits (Figures S1 and S2). Since no major differences between the three solvents options were obtained, ACN/water (50:50, *v*/*v*) was selected to match the solvents of the mobile phase.

**Figure 1:**
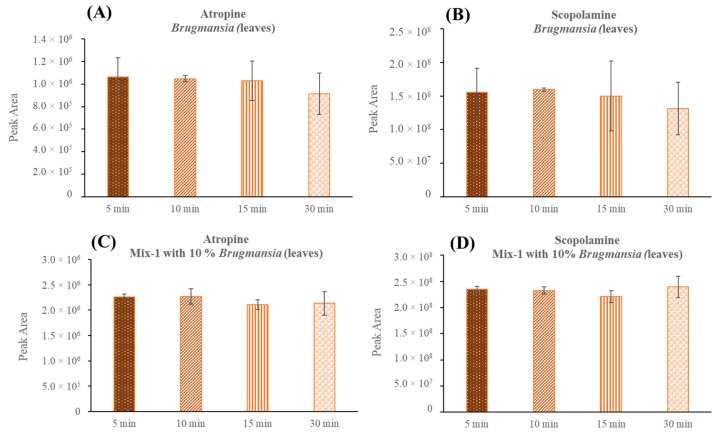
Peak area obtained for atropine and scopolamine at different extraction times in *Brugmansia versicolor* leaves (**A**,**B**) and mixed leafy vegetables (Mix-1) sample spiked with 10% *Brugmansia versicolor* leaves (**C**,**D**).

Finally, the extraction time on a shaker plate in real samples containing TAs was verified. For this, the μ-QuEChERS protocol was applied to the samples of the *Brugmansia versicolor* leaves, and agitation times of 5, 10, 15, and 30 min were tested (Figure 1A,B).

Before injection, the sample was reconstituted in 10 mL of ACN/water (50:50, *v*/*v*) and an aliquot was further diluted 10-fold with the same solvent, due to the high concentration of atropine and scopolamine in this plant. In parallel, this same test was then performed on a sample (Mix-1) intentionally contaminated with 10% *Brugmansia versicolor* leaves (Figure 1C,D). In this case, the sample was reconstituted in 500 µL of ACN/water (50:50, *v*/*v*), and a 50 µL aliquot was diluted in 1 mL. Figure 1 shows the results of the extraction time optimization. As can be observed, there were no differences between the times studied and, for this reason, 5 min was set as the optimum extraction time.

### 2.2. μ-QuEChERS Procedure Validation

The methodology based on μ-QuEChERS-HPLC-MS/MS was successfully validated, being the respective results shown in Table 2. Good linear regression was obtained for both analytes (being R^2^ ≥ 0.997), according to the criteria established in the validation guides [23,24]. A low dispersion between slopes was obtained with RSD ≤1.5% for both analytes. Regarding the matrix effect, atropine showed values of −38% and scopolamine of −39%. These values exceed +/− 20% according to the SANTE guide, indicating that there was signal suppression [24]. Therefore, to quantify the target analytes in the real samples, matrix-matched calibration curves had to be used to compensate for the errors associated with these matrix effects. Low detection limits were found for both analytes, being MQL 2.3 ng/g for atropine and 2.2 ng/g for scopolamine, and MDL 0.7 ng/g and 0.6 ng/g, respectively. Therefore, the proposed method shows lower detection limits than those proposed by EU recommendation 2015/976 [7], where the MQL should preferably be less than 5 ng/g and not exceed 10 ng/g in the case of agricultural products, ingredients, food supplements, and infusions. This demonstrates the good sensitivity of the method.

**Figure 2:**
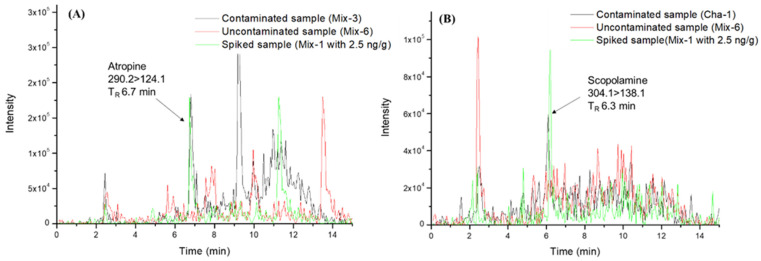
Chromatograms for atropine (**A**) and scopolamine (**B**) for their quantification ion in uncontaminated, contaminated, and spiked samples at the lowest level of the matrix-matched calibration curve (2.5 ng/g).

To assess selectivity, contaminated, uncontaminated, and spiked samples at the lowest concentration were compared to verify the absence of interfering peaks at the retention time (t_R_) of atropine and scopolamine, with an SD ≤ 0.1 min. Figure 2 shows the absence of interfering peaks in a t_R_ of ± 0.1 min in an uncontaminated sample (Mix-6) versus contaminated samples (Mix-3 and Cha-1) and spiked sample (Mix-1) with 2.5 ng/g. Also, ion transition ratios in unit mass resolution MS/MS were checked in a contaminated sample and compared to the spiked samples. The deviation was less than 30% (relative abundance), so the selectivity of the method is considered adequate.

Finally, accuracy and precision were evaluated at three levels. The lowest level (5 ng/g) was established based on the MQL recommended in legislation 2015/976 [7]. Table 2 shows the recoveries for accuracy at the three validated levels (5, 25 and 200 ng/g). Atropine showed recoveries between 90–100%, whereas scopolamine showed recoveries between 93–95%. According to the validation guidelines, the recovery values after method validation should be between 70% and 120%. Therefore, both analytes showed good recoveries at the three levels studied. Consequently, precision showed good RSD % at the three levels evaluated (Table 2). In intra-day precision, RSD (%) was ≤10% for atropine and ≤8% for scopolamine, and in inter-day precision, RSD (%) was ≤12% for atropine and ≤13% for scopolamine. The values obtained in Table 2 comply with the values recommended by the validation guides, RSD ≤ 20%.

### 2.3. Analysis of TA-Producing Plants and Contamination Assays

To carry out the contamination tests with real samples that naturally contain TAs, different samples (*Brugmansia versicolor*, *Solandra maxima* and *Convolvulus arvensis*) were analysed with the optimized and validated protocol. *Brugmansia versicolor* and *Solandra maxima* were collected on Madeira Island because they grow naturally throughout the island. For this analysis, six sample portions (*n* = 6) were taken to obtain a representative sample, since the concentrations of TAs vary between the part of the plant [25]. Table 3 shows the results obtained for the TA-producing plant samples analysed.

*Brugmansia versicolor* is the plant with the highest scopolamine content, with up to 1771 mg/kg found in the flower, whereas the leaves show a lower concentration of around 297 mg/kg. These data corroborate the EFSA compendium of botanicals [26], which states that the genus *Brugmansia* spp. generally contains scopolamine. Lower amounts have been found in *Solandra maxima*, again with the leaves containing lower concentrations of both TAs than the flowers (Table 3). According to the EFSA [26], this plant contains scopolamine and atropine, although atropine generally appears in the form of its more dangerous enantiomer (-)-hyoscyamine. *Convolvulus arvensis* was also analysed, since some studies point to the appearance of TAs in this plant [27]. In this case, the sample was collected in Spain, as it is widespread as an invasive climbing plant that grows as a weed among vegetable crops. In this plant, only small concentrations of atropine (0.0083 ± 0.0012 mg/kg) were found (*n* = 6).

After the analysis of the TA-producing plants, the developed methodology was further challenged in the analysis of intentionally contaminating a leafy vegetable sample (Mix-1) with 10% (*w*/*w*) of *Brugmansia versicolor* and *Solandra maxima* (flowers and leaves). *Convolvulus arvensis* was not included in the study because it contained only atropine, and only in low concentrations. The results of this study (Table 4) showed that the method is effective, since the recoveries obtained ranged between 82 and 110% for both the analytes studied.

### 2.4. Application of the μ-QuEChERS Procedure to Samples of Fresh Leafy Vegetables

Overall, eighteen samples of leafy vegetables were analysed in triplicate with the proposed methodology. To minimize signal suppression errors and obtain more accurate results (see Section 4.5), the ratios between the areas obtained for the target analytes and the internal standards ((±)-atropine-D3 or (-)-scopolamine-D3) were interpolated into matrix-matched calibration curves with the internal standards.

The results obtained are presented in Table 5. Atropine was detected in twelve samples, of which only three could be quantified (concentrations of 2.7 ng/g (Mix-5), 3.2 ng/g (Mix-3) and 3.4 ng/g (Mix-9)). The rest of the samples contained a concentration ≤MQL (2.3 ng/g). In turn, scopolamine was only detected in one sample, with a concentration below the ≤MQL (2.2 ng/g). The moisture content of the samples was taken into consideration to express the concentration of the target TAs as ng/g fresh weight. The humidity of the samples varied between 94–97%, except for Kal-1 (86%). For the samples with atropine contamination, the moisture content was 95% for Mix-3 and 94% for Mix-5 and Mix-9.

The samples Mix-3, Mix-5, and Mix-9 here reported with atropine presence above MQL are samples of different lettuces and other leafy vegetables, made up of iceberg lettuce, spinach sprouts, and arugula, among others (see Table 6 in Section 4.2). These types of samples of mixed leaf salads that had been cut, washed, and packaged for commercialization can be accidentally contaminated during vegetable harvesting or salad processing. This, however, does not explain the contamination of samples Ice-2, which was purchased as whole lettuce, and Cha-2, which was collected as whole Swiss chard from a vegetable patch. In these samples, there are no parts of other plants present, so contamination with TA-producing plants was not possible. In addition, as was indicated in Section 2.3, the presence of TAs in *Convolvulus arvensis*, one of the most noxious weeds of agricultural fields throughout temperate regions, is very low. For this reason, in the case of Ice-2 and Cha-2, the small amount of atropine detected can be a consequence of the horizontal and natural transference of TAs through the soil. This can occur among living plants growing nearby or even from dead plants, since the soil and compost employed in the pots of these plants had previously been used to grow other TA-producing plants. Moreover, taking into consideration that TAs are water-soluble compounds, rainwater can favor this hypothetic horizontal transference. A similar phenomenon has been proposed previously to explain the presence of toxic pyrrolizidine alkaloids in some aromatic herbs [28].

There are few works dealing with the presence of TAs in plant-based foods reported in the literature. For example, Mulder et al. [3] studied the occurrence of TAs in children’s foods as cereal-based, ready-to-eat meals containing vegetables, mixed vegetable stir-fry, and other mixed vegetable products (bell pepper, potato, courgette, onion, cauliflower, cabbage, peas, green beans, broccoli, and carrot). Of all the samples analysed, low concentrations of atropine and scopolamine were found between 0.1–1.25 ng/g in meals prepared for children. For the rest of the vegetable samples studied, only atropine and scopolamine were detected in some of the samples. In general, these samples presented higher concentrations of other non-legislated TAs, such as tropine or pseudotropine. More recently, Castilla-Fernandez et al. [6] found similar concentrations of atropine and scopolamine in spinach-based infant food products (between 0.02–0.06 ng/g) and frozen spinach samples (between 0.04–8.19 ng/g fresh weight).

The concentrations of TAs found in the leafy vegetable samples analysed in this work and in the studies mentioned above are below the concentrations legislated for other foods in the regulation EU 1408/2021 [4]. Even so, considering that there are no maximum limits established for the presence of TAs in leafy vegetable foodstuffs and that the few works reported so far point to contamination of these foods with atropine, scopolamine and other non-legislated TAs, it is becoming obvious that specific regulations should be adopted to control the presence of TAs in leafy vegetable foodstuffs.

## 3. Conclusions

This study proposes an analytical method involving miniaturized sample preparation with a µ-QuEChERS protocol combined with HPLC-MS/MS to determine atropine and scopolamine in leafy vegetable samples. This methodology proved to be a more environmentally friendly strategy by using a reduced amount of sample, solvents, and cleaning salts compared to the original QuEChERS protocol. The analytical methodology was successfully validated, demonstrating low limits and good accuracy and precision for both analytes. The performance of the method was also demonstrated after application to samples of TA-producing plants (*Brugmansia versicolor*, *Solandra maxima* and *Convolvulus arvensis*) and in a contamination assay with leafy vegetables contaminated with 10% of *B. versicolor* and *S. maxima*. This study showed recoveries close to 100%. In addition, the method was used to monitor the presence of atropine and scopolamine in eighteen samples of leafy vegetables, detecting atropine in twelve and scopolamine in one sample.

## 4. Materials and Methods

### 4.1. Chemicals, Reagents, and Standard Solutions

ACN and MeOH LC-MS-grade salts used in the μ-QuEChERS procedure, such as anhydrous magnesium sulphate (MgSO_4_), sodium chloride (NaCl), sodium citrate tribasic dihydrate (C_6_H_5_Na_3_O_7_ 2 H_2_O), disodium hydrogen citrate sesquihydrate (C_6_H_6_Na_2_O_7_ 1.5 H_2_O), and PSA, were obtained from Scharlab (Barcelona, Spain). FA (purity ≥ 99% Optima™), LC-MS grade, was acquired from Fisher Chemical (Madrid, Spain). Ultrapure deionized water (18.2 MΩ cm quality) used for mobile phases and aqueous solutions was obtained using a Millipore Milli-Q-System (Billerica, MA, USA). Nylon syringe filters (0.45 µm, 13 mm) used to filter the samples before analysis by HPLC-MS/MS were purchased from Mervilab (Madrid, Spain).

The internal standard of (-)-scopolamine-D3 hydrochloride solution (100 µg/mL in ACN:Water (9:1), MW 342.83 g/mol; CAS 1202357-61-6) and analytical standards of atropine sulphate (≥99%, MW 289.37 g/mol CAS 51-55-8) and scopolamine hydrobromide (≥98%, CAS 6533-68-2) were acquired from Sigma-Aldrich (St. Louis, MO, USA). The internal standard of (±)-atropine-D3 (1 mg, MW 292.39 g/mol) was purchased from Análisis Vínicos (Tomelloso, Spain). Individual stock standard solutions of atropine and scopolamine were prepared at 1000 µg/mL in MeOH. (±)-atropine-D3 was prepared in 10 mL of MeOH (100 µg/mL) and (-)-scopolamine-D3 hydrochloride solution (100 µg/mL) was diluted in 10 mL of MeOH (10 µg/mL). Working standard solutions were prepared by appropriate dilution at the desired concentration in ACN/Water (50:50, *v*/*v*). All prepared solutions were stored in the dark at −20 °C.

### 4.2. Samples

A total of eighteen samples of leafy vegetables were collected to carry out this work (Table 6). To get a representative sample, different samples from the same batch were purchased at local supermarkets in Madrid (Spain), except for the whole Swiss chard sample (Cha-2) that was collected from a vegetable patch in Madrid.

Eleven samples were mixtures of different lettuces and other leafy vegetables (samples Mix-1 to Mix-11). Seven samples contained only a single leafy vegetable. On the other hand, samples of TA-producing plants were collected on Madeira Island (Portugal, leaves and flowers of *Brugmansia versicolor* and *Solandra maxima*) and in Madrid (leaves, stems and flowers of *Convolvulus arvensis*). To preserve and achieve homogeneous samples, the samples were lyophilized for 24–48 h in a LyoBench −55 °C laboratory freeze-dryer (Noxair Life Sciences S.L., Barcelona, Spain). Next, all samples were ground with an A11 basic analytical mill (IKA, Staufen, Germany), homogenized, and sieved to obtain the same particle size. Afterwards, the samples were stored in the dark in a desiccator until their use.

### 4.3. μ-QuEChERS Procedure

**Figure 3:**
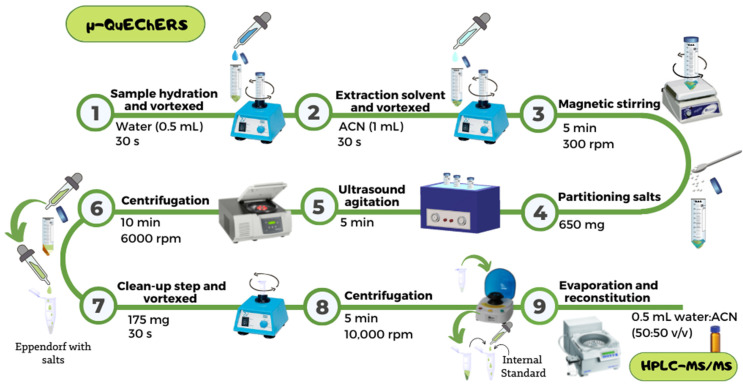
Schematic diagram of the μ-QuEChERS methodology used for the determination of atropine and scopolamine.

The samples were extracted through a miniaturized QuEChERS based on the original methodology proposed by Anastassiades et al. [14]. To carry out the protocol (Figure 3), 0.1 g of the lyophilized sample was weighed on an analytical balance with a resolution of 0.001 g. Subsequently, the weighed sample was mixed with 0.5 mL of water and vortexed for 30 s. Then, 1 mL of ACN was added and the mixture vortexed for 30 s and shaken on a shaker plate for 5 min at 300 rpm. After this time, 0.65 g of the partitioning salts mixture formed by MgSO_4_, NaCl, C_6_H_5_Na_3_O_7_ 2 H_2_O, and C_6_H_6_Na_2_O_7_ 1.5 H_2_O, in proportions of 4:1:1:0.5, was added.

The mixture was vortexed for 30 s, followed by 5 min of ultrasound agitation and 10 min of centrifugation at 6000 rpm in a centrifuge Digicen 21 R from Ortoalresa (Madrid, Spain). After centrifugation, the upper part of the extract was transferred into an Eppendorf^®^ containing MgSO_4_ (150 mg) and PSA (25 mg) for the clean-up step. The new mixture was vortexed for 30 s and centrifuged for 5 min at 10,000 rpm in a mini centrifuge model 2507/14 from Nahita (China). Next, an aliquot of 10 µL of the 500 ng/mL internal standards solution containing (±)-atropine-D3 and (-)-scopolamine-D3 was added to the supernatant obtained. Later, the supernatant was evaporated to dryness in an Eppendorf^®^ Concentrator Plus from Eppendorf SE (Hamburg, Germany) and redissolved in 500 µL of ACN/Water (50:50, *v/v*). The eluate obtained was filtered before the analysis in the chromatography system using a 0.45 μm nylon syringe filter (13 mm diameter).

### 4.4. HPLC-MS/MS Conditions for Analysis of Atropine and Scopolamine

Detection and quantification of atropine and scopolamine was carried out with a Varian 1200/1200 L LC-MS/MS (Varian Ibérica, Spain). The Varian Prostar HPLC was equipped with a ProStar 410 autosampler (100 µL loop), two ProStar 210/215 solvent delivery modules, and a thermostatic compartment for the chromatographic column. The HPLC was coupled with a triple quadrupole mass spectrometer detector (1200 L TQ) with electrospray ionization (ESI) ion source (data acquisition system MS Workstation version 6.3). The analytical separation was achieved using a reverse phase column C18 Kromaphase 100 column (150 mm × 2.0 mm, 3.5 μm particle size) with a C18 Kromaphase guard column (10 mm × 4.0 mm I.D., 5 μm particle size) at 30 °C, acquired from Scharlab (Barcelona, Spain). The injection volume was 10 µL (partial injection) and the separation occurred in gradient mode with a flow rate of 0.25 mL/min. The mobile phases and the gradient elution were similar to those of our previous work [29], consisting of solvent A (Milli-Q water) and solvent B (ACN), both containing 0.1% formic acid. The gradient used started at 90% A and then decreased linearly to 30% in 10 min and returned to 90% in 1 min, holding these conditions for 4 min. The total run-time was 15 min.

Data acquisition in the mass spectrometry detector was performed using an ESI source operating in positive mode. The conditions established in the MS detector were set at 350 °C and 22 psi for N_2_ used as drying gas, 58 psi for N_2_ used as nebulizer gas pressure, 5000 V for the capillary voltage, 600 V for the shield, 1.90 mTorr for Argon used as collision gas, and 1535 V for detector voltage. For the detection of the analytes, the multiple reaction monitoring (MRM) mode was used (mass peak width Q1 2.5; mass peak width Q3 2.5; scan width in MRM 0.70), and the analytes were monitored at a cone voltage of 70 V. Table 7 shows mass spectrum parameters, product ions, and ions used for quantification of atropine, scopolamine, (±)-atropine-D3, and (-)-scopolamine-D3.

### 4.5. Samples Quantification

For the quantification of atropine and scopolamine, the samples were analyzed in triplicate (*n* = 3) with the validated methodology, according to Section 4.6. Matrix-matched calibration curves were prepared at seven calibration points, with concentrations ranging from 0.5 to 500 ng/mL, using Mix-1 as a representative sample. To each point of the matrix-matched calibration curves, an aliquot of 10 µL of 500 ng/mL solution containing (±)-atropine-D3 and (-)-scopolamine-D3 was added. Curves were constructed by plotting the ratio of the analyte peak area to the internal standard peak area versus the analyte concentration. The quotient areas obtained after the analysis of the samples were interpolated on the matrix-matched calibration curve. The mean and standard deviation of the concentrations obtained were then calculated.

### 4.6. Method Validation

The proposed methodology was validated in terms of linearity, matrix effects, method detection limits (MDLs) and quantification limits (MQLs), selectivity, accuracy, and precision. For this, the recommendations of different validation guides, such as Guidance SANTE/11312/2021 for pesticides [24], IUPAC harmonized guidelines for single-laboratory validation [30], and AOAC International Guidelines for Dietary Supplements and Botanicals [23] were followed since there are no specific recommendations for the validation of methods applied to TAs.

Linearity was assessed by constructing a matrix-matched calibration curve with an internal standard for each analyte. Curves were prepared on three consecutive days and at seven calibration points with concentrations ranging from 0.5 to 500 ng/mL. To carry out the calibration curve, a representative sample (Mix-1) was selected and the μ-QuEChERS procedure was applied to the lyophilized sample. The extracts obtained after the protocol were spiked at the appropriate concentration level of the calibration curve with an aliquot of the standard solution containing both TAs. To these extracts, an aliquot of 10 µL of the 500 ng/mL containing (±)-atropine-D3 and (-)-scopolamine-D3 internal standards was added to each point of the matrix-matched calibration curve to correct for the matrix effect in each analyte. At the same time, a blank sample (non-spiked sample) was prepared for the correction of the signal in case of contamination by some analyte. Linearity was evaluated through the correlation coefficient (R^2^) obtained by plotting the calibration curve by dividing the area of atropine and scopolamine by the area of the internal standard versus the concentrations of the analytes. Matrix effects were verified by comparing the slope of the matrix-matched calibration curve with the slope of the solvent calibration curve by applying the equation ((slope matrix-matched/slope solvent)-1) × 100, both expressed in ng/mL [24,31]. The sensitivity of the method was calculated from the MDL and MQL as three and ten times, respectively, the S/N of the response obtained in HPLC-MS/MS at the lowest concentration of the matrix-matched calibration curve (0.5 ng/mL). The selectivity of the method was checked at the characteristic t_R_ for atropine and scopolamine with an uncontaminated sample, a contaminated sample, and a spiked sample at the lowest calibrated level. Also, the ion transition ratios in unit mass resolution were verified. Accuracy was evaluated in terms of recoveries for three concentration levels, 5 ng/g (low), 25 ng/g (medium), and 200 ng/g (high). For this, six portions of Mix-1 (*n* = 6) were spiked with (±)-atropine-D3 and (-)-scopolamine-D3 internal standards of each level on the lyophilized sample, at the beginning of the protocol, and one was spiked at the end (simulated sample) to estimate the recovery. In turn, precision was evaluated as intra- and inter-day precision at three concentration levels and expressed as relative standard deviation (RSD %). Intraday precision was carried out by analyzing six replicates (*n* = 6) in one day, spiked at three concentration levels. The inter-day precision was estimated by analyzing three different replicates spiked at three concentration levels (*n* = 9) on three different days. In all cases, the extracts were injected into HPLC-MS/MS in triplicate.

## Figures and Tables

**Table 1:** Recovery percentages applying the µ-QuEChERS procedure to a fresh sample versus a hydrated lyophilized sample with different amounts of water.

Type of Sample ^a^	Sample Amount (g)	Water Amount (mL) ^b^	Spiked Level (ng/g) ^c^	Atropine Recovery ± SD (%)	Scopolamine Recovery ± SD (%)
Fresh	1	-	5	73 ± 3	77 ± 9
Lyophilized	0.1	0.9	5	40 ± 4	62 ± 17
Lyophilized	0.1	0.75	5	95 ± 11	101 ± 18
Lyophilized	0.1	0.5	5	98 ± 5	95 ± 2
Lyophilized	0.1	0.5	100	84 ± 1	105 ± 13

^a^ Mix-1 sample was used for this study; ^b^ mL of water for sample hydration; ^c^ spiked with a solution containing atropine and scopolamine. See details in Section 4.3 and Section 4.4.

**Table 2:** Validation data of the proposed method based on the μ-QuEChERS procedure followed by HPLC-MS/MS.

Analyte	Linear Range ^a^ (ng/mL)	Linearity R^2^ Slope RSD (%)	MDL ^b^ (ng/g)	MQL ^c^ (ng/g)	ME ^d^ (%)	Spiked Level (ng/g)	Accuracy *n* = 6 (Recovery ± SD %)	Intra-Day Precision *n* = 6, 1 Day (RSD %)	Inter-Day Precision *n* = 9, 3 Days (RSD %)
		0.06*x* + 0.27				5	90 ± 10	9	11
Atropine	0.5–500	0.998	0.7	2.3	−38	25	100 ± 10	10	12
		1.5				200	96 ± 4	4	11
		0.13*x* + 0.88				5	93 ± 7	8	13
Scopolamine	0.5–500	0.997	0.6	2.2	−39	25	96 ± 5	5	10
		0.3				200	95 ± 6	6	7

^a^ Linear range expressed in weight/weight corresponds to 2.5–2500 ng/g according to the validated analytical methodology. ^b^ MDL: Method detection limit. ^c^ MQL: Method quantification limit. ^d^ Matrix effect (ME). Matrix-matched calibration for atropine y = 1.11∙×10^6^ *x* +5.0 × 10^6^ and scopolamine y = 5.16×10^5^ *x* +2.9 × 10^6^. Solvent-based calibration for atropine y = 1.79 × 10^6^ *x* −2.2 × 10^6^ and scopolamine y = 8.41 × 10^5^ *x* − 1.5 × 10^6^.

**Table 3:** Atropine and scopolamine content in TA-producing plants.

TA-Producing Plant		Family	Sample Analysed	Atropine (mg/kg)	Scopolamine (mg/kg)
*Solandra maxima* ^a^	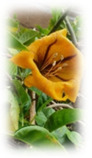	Solanaceae	Flowers	10.4 ± 0.1	0.11 ± 0.05
			Leaves	0.20 ± 0.07	0.023 ± 0.006
*Brugmansia versicolor* ^a^	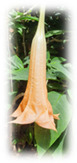	Solanaceae	Flowers	5 ± 1	1771 ± 167
			Leaves	0.9 ± 0.3	297 ± 36
*Convolvulus arvensis* ^b^	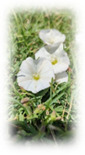	Convolvulaceae	Leaves, stems and flowers	0.0083 ± 0.0012	^c^ ND

^a^ Collected on Madeira Island. ^b^ Collected in Spain. ^c^ ND: not detected.

**Table 4:** Atropine and scopolamine recovery obtained after spiking a sample of leafy vegetables (Mix-1) with 10% (*w*/*w*) of TA-producing plants.

	Atropine			Scopolamine		
Contaminated Sample	Expected Content (ng/g)	Found Content (ng/g ± SD)	Recovery (% ± SD)	Expected Content (ng/g)	Found Content (ng/g ± SD)	Recovery (% ± SD)
Mix-1 with 10% *S. maxima* flowers	1043	874 ± 56	84 ± 5	11	11 ± 1	102 ± 9
Mix-1 with 10% *S. maxima* leaves	20	20 ± 2	98 ± 9	2	2.2 ± 0.5	110 ± 3
Mix-1 with 10% *B. versicolor* flowers	544	444 ± 19	82 ± 3	177,067	156,468 ± 8776	88 ± 7
Mix-1 with 10% *B. versicolor* leaves	89	92 ± 3	104 ± 3	29,743	29,576 ± 3163	99 ± 11

**Table 5:** Atropine and scopolamine content (ng/g) in leafy vegetable samples.

Sample	Atropine (ng/g ± SD)	Atropine (ng/g Fresh Weight ± SD) ^a^	Scopolamine (ng/g ± SD)	Scopolamine (ng/g Fresh Weight ± SD)
Mix-1	≤MQL	-	ND	-
Mix-2	≤MQL	-	ND	-
Mix-3	3.2 ± 1.9	0.16 ± 0.09	ND	-
Mix-4	≤MQL	-	ND	-
Mix-5	2.7 ± 0.9	0.16 ± 0.05	ND	-
Mix-6	ND	-	ND	-
Mix-7	ND	-	ND	-
Mix-8	≤MQL	-	ND	-
Mix-9	3.4 ± 0.8	0.20 ± 0.05	ND	-
Mix-10	ND	-	ND	-
Mix-11	ND	-	ND	-
Ice-1	≤MQL	-	ND	-
Ice-2	≤MQL	-	ND	-
Kal-1	≤MQL	-	ND	-
Spi-1	ND	-	ND	-
Cha-1	≤MQL	-	≤MQL	-
Cha-2	≤MQL	-	ND	-
Aru-1	ND	-	ND	-

ND: Not detected. ≤MQL: below or equal to the limit of quantification of the method (atropine MQL = 2.3 ng/g and MDL = 0.7 ng/g; scopolamine MQL = 2.2 ng/g and MDL = 0.6 ng/g). ^a^ ng/g fresh weigh = [ng/g lyophilized × (100 − M)]/100. Moisture (M) Mix-3 = 95%; M Mix-5 = 94%; M Mix-9 = 94%.

**Table 6:** Leafy vegetables analysed in the work.

Code	Sample Description	Origin	Ingredients
Mix-1	Mixed leafy vegetables	Local supermarket	Curly escarole, red cabbage, lollo rosso lettuce, spinach sprouts and arugula
Mix-2	Mixed leafy vegetables	Local supermarket	Curly escarole, red cabbage, lollo rosso lettuce, spinach sprouts and arugula
Mix-3	Mixed leafy vegetables	Local supermarket	Iceberg lettuce, carrot and red cabbage
Mix-4	Mixed leafy vegetables	Local supermarket	Green sprout lettuce and red sprout lettuce
Mix-5	Mixed leafy vegetables	Local supermarket	Curly endive, red lettuce sprouts, red radicchio and mizuna
Mix-6	Mixed leafy vegetables	Local supermarket	Green lettuce sprouts, red lettuce sprouts and wild arugula
Mix-7	Mixed leafy vegetables	Local supermarket	Arugula and lamb’s lettuce
Mix-8	Mixed leafy vegetables	Local supermarket	Red lettuce sprouts, lamb’s lettuce, arugula and watercress
Mix-9	Mixed leafy vegetables	Local supermarket	Red lettuce sprouts, spinach sprouts, green lettuce sprouts, lamb’s lettuce, arugula and tatsoi
Mix-10	Mixed leafy vegetables	Local supermarket	Green baby leaves, red baby leaves and lamb’s lettuce
Mix-11	Mixed leafy vegetables	Local supermarket	Spinach sprouts, red spinach and arugula
Ice-1	Chopped iceberg lettuce	Local supermarket	Iceberg lettuce (*Lactuca sativa var. capitata*)
Ice-2	Whole iceberg lettuce	Local supermarket	Iceberg lettuce (*Lactuca sativa var. capitata*)
Kal-1	Chopped kale	Local supermarket	Green curly kale (*Brassica oleracea var. sabellica* L.)
Spi-1	Spinach sprouts	Local supermarket	Spinach sprouts (*Spinacia oleracea* L.)
Cha-1	Chopped Swiss chard	Local supermarket	Swiss chard (*Beta vulgaris var. cycla*)
Cha-2	Whole Swiss chard	Vegetable patch	Swiss chard (*Beta vulgaris var. cycla*)
Aru-1	Arugula salad	Local supermarket	Arugula (*Eruca vesicaria*)

**Table 7:** Parameters of mass spectrometry analysis.

Analyte	t_R_ (min)	Ionization Mode	Precursor Ions (Q_1_, m/z, [M+H]+)	Capillary (V)	MS^2^ Product Ions (Q_3_, m/z)	CE (V)	Dwell Time (s)	Chemical Structures and Fragment Ions
Atropine	6.7	ESI (+)	290.2	70	90.9	34	0.25	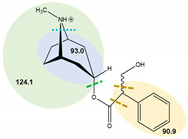
					93.0	29	0.25	
					124.1 *	20.5	0.25	
(±)-Atropine-D3	6.7	ESI (+)	293.4	70	92.9	25	0.25	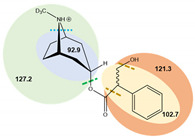
					102.7	42	0.25	
					121.3	28	0.25	
					127.2 *	21	0.25	
Scopolamine	6.3	ESI (+)	304.1	70	121.0	16	0.25	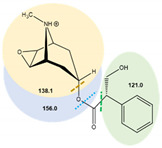
					138.1 *	12	0.25	
					156.0	9.5	0.25	
(-)-Scopolamine-D3	6.3	ESI (+)	307.4	70	121.1	24.5	0.25	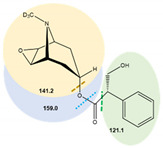
					141.2 *	24.5	0.25	
					159.0	9	0.25	

* Ions used for quantification.

## Data Availability

Not applicable.

## Supplementary Materials

The following supporting information can be downloaded at: https://www.mdpi.com/article/10.3390/toxins14100650/s1, Figure S1: Chromatograms for a sample (Mix-1) spiked with 5 ng/g of atropine extracted and purified with the μ-QuEChERS protocol and injected in different reconstitution solvents. **A**: ACN; B: ACN-Water (50/50, *v*/*v*); **C**: Water (0.1% FA); **D**: ACN-Water (both with 0.1%, FA 10/90, *v*/*v*); **E**: MeOH; **F**: MeOH-Water (50/50, *v*/*v*); **G**: Water.; Figure S2: Chromatograms for a sample (Mix-1) spiked with 5 ng/g of scopolamine extracted and purified with the μ-QuEChERS protocol and injected in different reconstitution solvents. **A**: ACN; B: ACN-Water (50/50, *v*/*v*); **C**: Water (0.1% FA); **D**: ACN-Water (both with 0.1%, FA 10/90, *v*/*v*); **E**: MeOH; **F**: MeOH-Water (50/50, *v*/*v*); **G**: Water.
