# Supplementary material for: Improved Analytical Approach for Determination of Tropane Alkaloids in Leafy Vegetables Based on µ-QuEChERS Combined with HPLC-MS/MS
Source: Toxins (Basel). 2022 Sep 20;14(10):650. doi: 10.3390/toxins14100650 (PMC9612249; doi:10.3390/toxins14100650)
Supplement: Supplementary file 1 [file toxins-14-00650-s001.zip › toxins-1899431-supplementary.pdf]

Supplementary Materials: Improved Analytical Approach for Determination of Tropane Alkaloids in Leafy Vegetables Based on μ-QuEChERS Combined with HPLC-MS/MS

Lorena González-Gómez, Sonia Morante-Zarcero, Jorge A.M. Pereira, José S. Câmara and Isabel Sierra

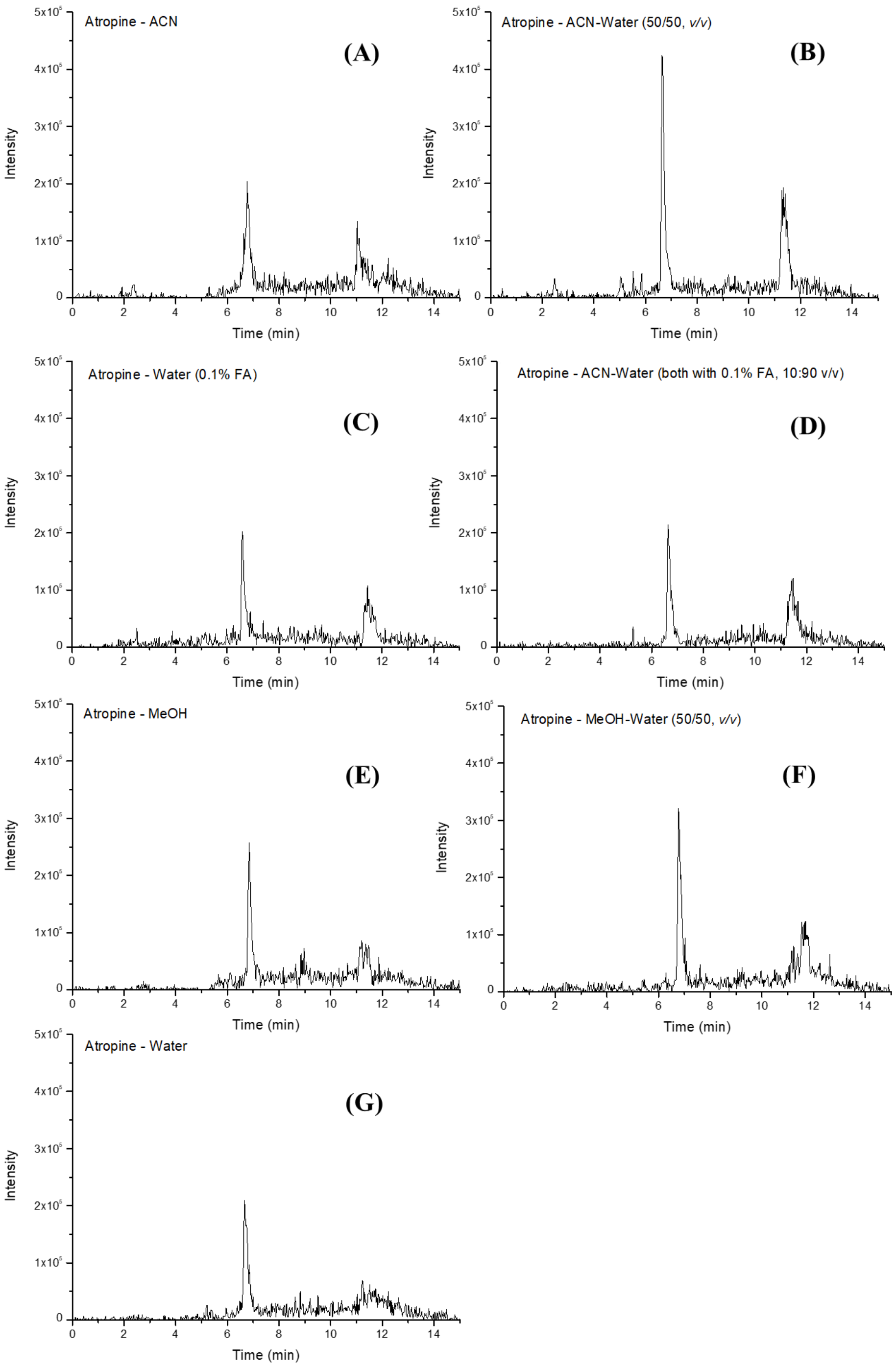

Figure S1. Chromatograms for a sample (Mix-1) spiked with 5 ng/g of atropine extracted and purified with the μ-QuEChERS protocol and injected in different reconstitution solvents. **A**: ACN; B: ACN-Water (50/50, *v/v*); **C**: Water (0.1% FA); **D**: ACN-Water (both with 0.1%, FA 10/90, *v/v*); **E**: MeOH; **F**: MeOH-Water (50/50, *v/v*); **G**: Water.

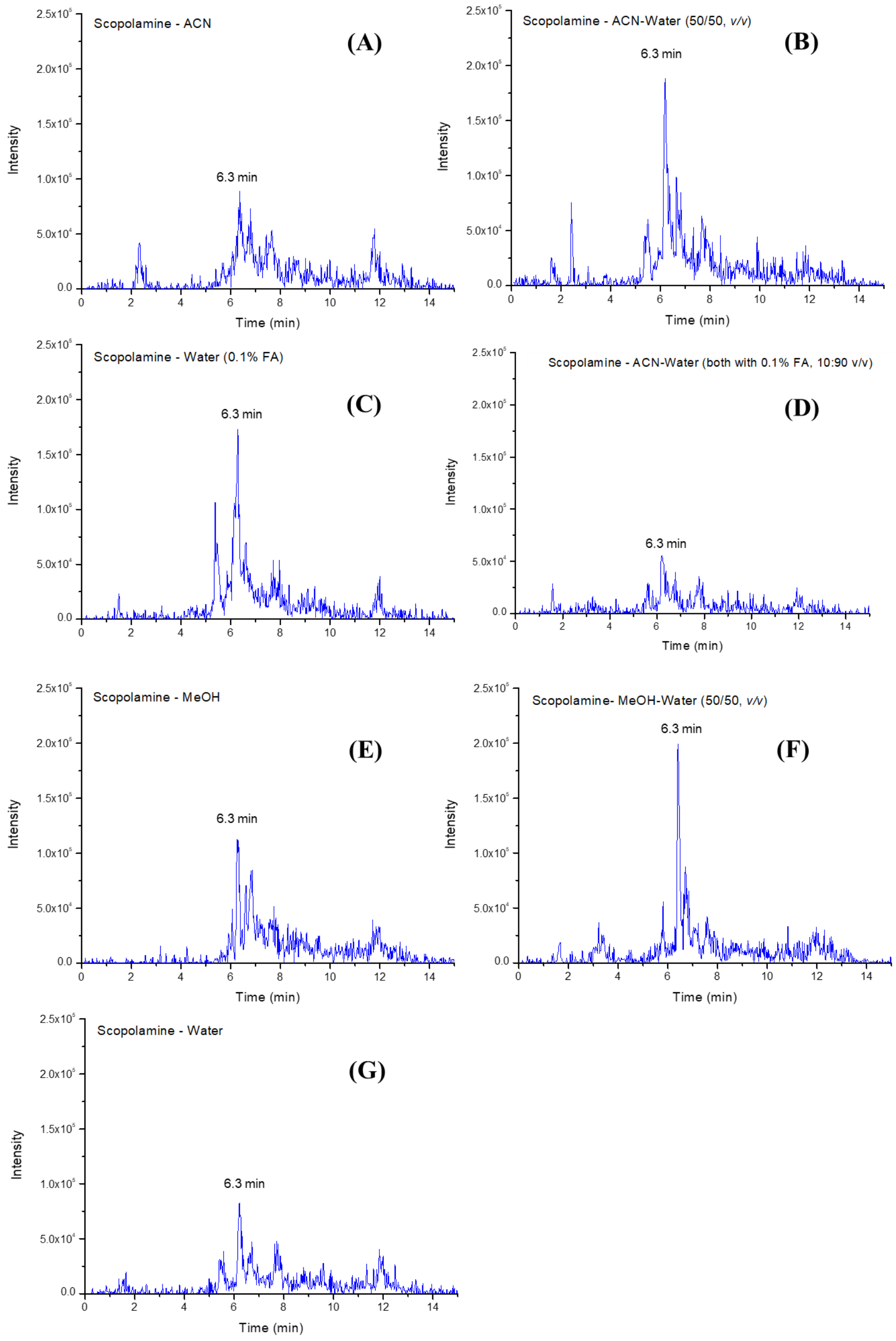

Figure S2. Chromatograms for a sample (Mix-1) spiked with 5 ng/g of scopolamine extracted and purified with the μ-QuEChERS protocol and injected in different reconstitution solvents. **A**: ACN; B: ACN-Water (50/50,

v/*v*); **C**: Water (0.1% FA); **D**: ACN-Water (both with 0.1%, FA 10/90, *v*/*v*); **E**: MeOH; **F**: MeOH-Water (50/50, *v*/*v*); **G**: Water.
